# Nitrogen fertilizer dose alters fungal communities in sugarcane soil and rhizosphere

**DOI:** 10.1038/srep08678

**Published:** 2015-03-02

**Authors:** Chanyarat Paungfoo-Lonhienne, Yun Kit Yeoh, Naga Rup Pinaki Kasinadhuni, Thierry G. A. Lonhienne, Nicole Robinson, Philip Hugenholtz, Mark A. Ragan, Susanne Schmidt

**Affiliations:** 1 School of Agriculture and Food Sciences, The University of Queensland, St. Lucia QLD, 4072, Australia; 2 Institute for Molecular Bioscience, The University of Queensland, St. Lucia QLD, 4072, Australia; 3 Australian Centre for Ecogenomics, School of Chemistry and Molecular Biosciences, The University of Queensland, St. Lucia QLD, 4072, Australia; 4 Australian Genome Research Facility Ltd, The University of Queensland, St. Lucia QLD, 4072, Australia; 5 School of Chemistry and Molecular Biosciences, The University of Queensland, St. Lucia QLD, 4072, Australia

## Abstract

Fungi play important roles as decomposers, plant symbionts and pathogens in soils. The structure of fungal communities in the rhizosphere is the result of complex interactions among selection factors that may favour beneficial or detrimental relationships. Using culture-independent fungal community profiling, we have investigated the effects of nitrogen fertilizer dosage on fungal communities in soil and rhizosphere of field-grown sugarcane. The results show that the concentration of nitrogen fertilizer strongly modifies the composition but not the taxon richness of fungal communities in soil and rhizosphere. Increased nitrogen fertilizer dosage has a potential negative impact on carbon cycling in soil and promotes fungal genera with known pathogenic traits, uncovering a negative effect of intensive fertilization.

The rhizosphere, the interface between soil and roots, is a biologically active zone where roots and microorganisms interact^1^. The identity of microorganisms in the rhizosphere has great influence on plant vigour and growth with beneficial, neutral and detrimental microbial relationships^2^. Structural and functional diversity of rhizosphere microbial populations in natural and agricultural systems is affected by plant species, crop cultivar, phenology, soil type, agronomy practice and other environmental factors^3^,^4^,^5^,^6^.

It is estimated that global N fertilizer use will increase threefold by 2050 to meet the growing need for food^7^. The use of chemical fertilizers is often accompanied by inefficiences that result in pollution and soil degradation^8^. The type and quantity of N fertilizer affects physical, chemical and biochemical properties of soil^9^,^10^, as well as bacterial and arbuscular mycorrhizal fungal (AMF) communities in the rhizosphere^11^,^12^.

Increasing the dose of NPK fertilizer has been associated with an increased presence of bacteria and fungi in crop soils^13^. However, whether the dose of fertilizer modifies rhizosphere fungal communities is largely unknown, with the exception of AMF which diminish in taxon richness in maize roots in response to N fertilizer^11^. The objective of this study was to investigate how N fertilizer rates influence fungal communities in rhizosphere and soil of a commercial sugarcane crop system.

## Results and Discussion

**Figure 1:**
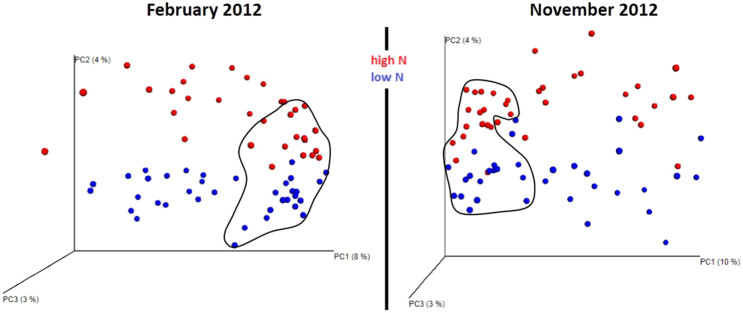
Beta diversity metrics based on fungal ITS1 and ITS2 sequences reveal distinctly clustered rhizosphere and soil (soil data are circled in black) fungal communities structured by nitrogen fertilizer application. Beta diversity fungal community clustering is observed for non-phylogenetic methods (binary Chord). The percentage of variation explained by the plotted principal coordinates is indicated on the axes.

We sampled a total of 822 operational taxonomic units (OTUs) (February) and 820 OTUs (November) of fungi from 1135428 (February) and 1187048 (November) pyrosequence reads, respectively. Irrespective of collection time, the Chao1 metric showed no difference in species richness between low and high N treatment: in February 18.6 ± 8.83 (high N) and 17.2 ± 9.72 (low N); in November 19.3 ± 8.57 (high N) and 18.7 ± 9.63 (low N). Similar to the Chao1 metric, Simpson's index confirmed no difference in species richness: in February 0.768 ± 0.162 (high N) and 0.773 ± 0.125 (low N); in November 0.787 ± 0.126 (high N) and 0.782 ± 0.133 (low N). However, while fungal taxon richness did not differ between N-fertilizer doses, clear differences were detected in the fungal community composition in sugarcane rhizosphere and soil at each sampling time (Fig. 1).

Sequence-based community profiling is increasingly adopted to study plant root-associated bacterial and fungal communities^14^,^15^ as this approach circumvents culture bottlenecks. Species richness can be estimated from read counts of the internal transcribed spacer (ITS) region, but in the case of fungal communities these estimates may be biased due to the differing lengths of the ITS in different fungal species^16^, as shorter regions are preferentially amplified^17^. However, this bias does not significantly alter estimates of relative abundance (species evenness) of the dominant OTUs^17^,^18^ and is expected to be even further attenuated in comparisons of relative abundance within a community, *e.g.* between treatments. Our results (Fig. 1), based on the relative abundance of dominant OTUs and showing compositional dissimilarities between sugarcane soil and rhizosphere fungal communities associated with N fertilizer dose, are therefore unlikely to be significantly affected by this amplification bias, and detected community dissimilarities would reflect true biological variation.

**Figure 2:**
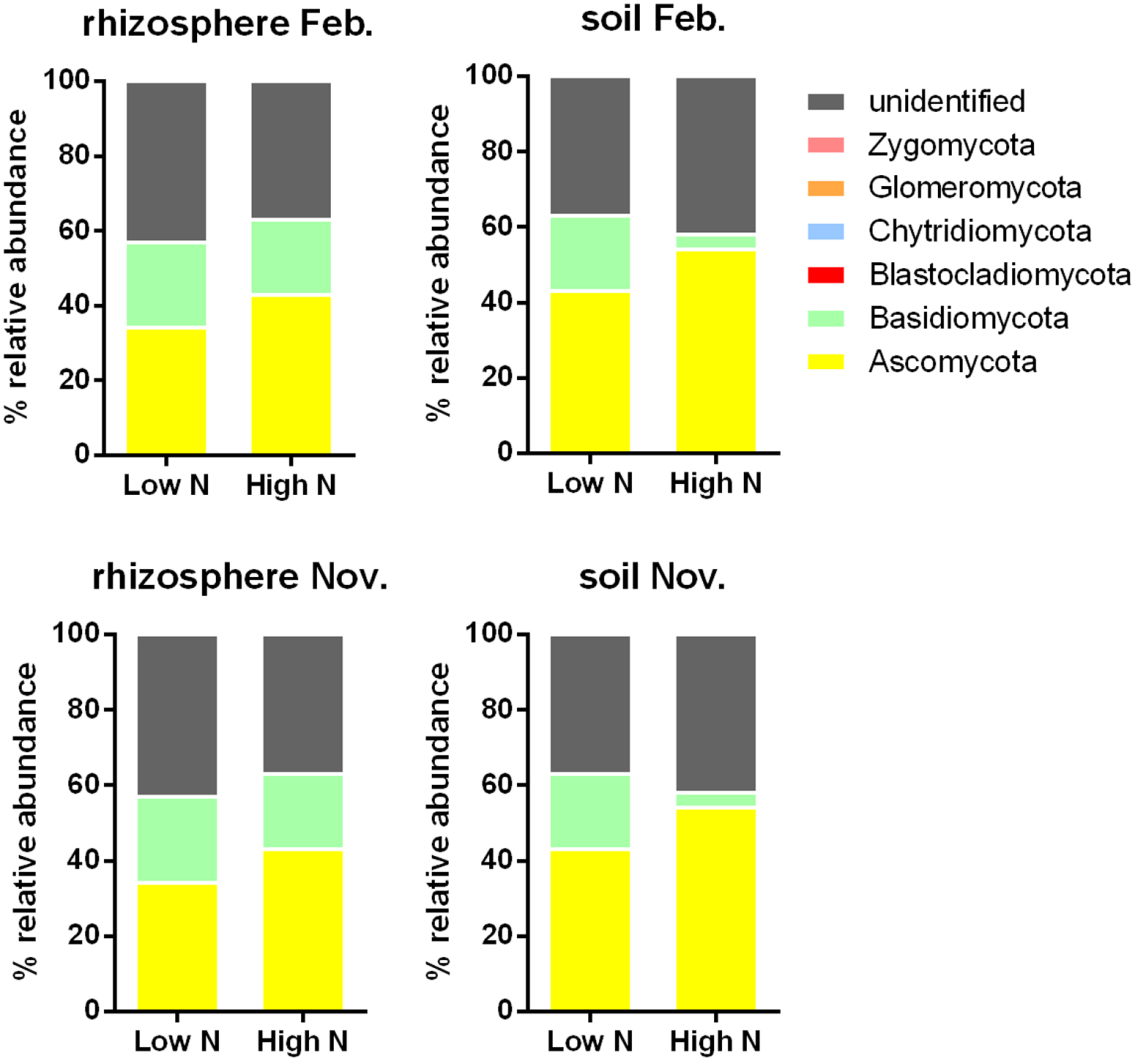
Effect of nitrogen fertilizer doses on fungal taxonomic diversity in sugarcane rhizosphere and soil. The samples were collected at two time points (February and November 2012).

Interesting results are apparent even at the phylum level. Across all soil and rhizosphere samples collected in February and November, fungal taxonomic diversity involves mainly two phyla, Ascomycota and Basidiomycota (Fig. 2). The relative abundance of Ascomycota was generally higher in high N fertilizer dose conditions compared to low N, whereas for Basidiomycota it was lower. Consistent with this result, most saprotrophic microfungi are Ascomycota^19^ and their growth rate is correlated with N availability^20^. Basidiomycetes are widely recognised as lignin decomposers^21^ and thus important for carbon cycling in soil; in the same way, this beneficial function could be adversely affected by high N dose (Fig. 2). In agreement to our results, deleterious effects of mineral fertilizers on soil and plant function has been proposed because it negatively impacts on symbiotic relationships, including diazotrophic^22^ and AM symbioses^23^,^24^.

To identify the known fungal genera that were most-altered in relative abundance by N fertilizer doses, we compared the relative abundances of identified OTUs in rhizosphere and soil between low N and high N doses, using the ratio (low N/high N) as a means of evaluation (Tables 1 and 2). The data show that in all samples, the genera positively or negatively affected include groups known for their positive impact on soil and plant health (biocontrol, decomposers) or to the contrary, for their negative impact as plant pathogens. For example, irrespective of collection time, the relative abundance of OTUs corresponding to the ascomycete genus *Clonostachys* and to the basidiomycete genus *Resinicium* were amongst the most promoted in rhizosphere and soil by low N doses (Tables 1 and 2). Both genera contain species with known antagonistic effects against other microorganisms^25^,^26^ and are therefore of interest as potential biological control agents against pathogens. Fungal genera positive for plant health were also promoted by increased N dose, however to a lesser extent. There was a tendency for high N to substantially increase the proportion of pathogenic genera (Table 1 and 2). Whereas higher doses of NPK fertilizer have been associated with increased biomass of fungi in soils of crop systems^13^, this result indicates that in sugarcane rhizosphere and soil, increased N fertilizer also modifies the composition of the fungal communities and, by promoting pathogenic fungi, may have a negative impact on plant health.

In summary, our findings add to understanding on how different doses of N influence fungal communities. The data show that the changes in relative abundance of fungal population in response to N doses are not restricted to AMF^11^ but span a wide range of fungal taxa, including genera known to influence plant health. Further research should elucidate the specific roles of these fungal taxa in sugarcane rhizosphere and soils, and on the heath of the plant. It is an attractive concept to manipulate the microbial community in the rhizosphere to reduce the need for agrochemicals, reduce disease incidence and improve crop performance^27^,^28^,^29^. To advance the ecological management of crop soils, understanding is needed of how beneficial microbial relationships can be fostered.

## Methods

### Sample collection

We sampled three individual plots within a 4-hectare field trial in the Burdekin region, Australia (near Ayr, S19°43.955′, E14°710.727′, 26 m above sea level). The soil is a silty-clay loam. Within each plot, half the sugarcane crops received an N supply rate of 200 kg N ha^−1^ y^−1^ (recommended ‘high’ N in the form of urea) while the other half received 40 kg N ha^−1^ y^−1^ (‘low’ N). From the three replicate plots, six bulk-soil and six roots with adhering soil (constituting the rhizosphere samples in our study) biological replicates were sampled at 0–10 cm depth from sugarcane receiving either N supply rate. Sampling was performed on a first and second ratoon crop of three sugarcane cultivars (Australian cultivars Q208 and Q186, and Brazilian cultivar SP79-2313) for a total of 144 root and bulk soil samples. Samples were immediately placed in a cool box for 2 days during transport to the laboratory and stored at −20°C for isolation of DNA. Sampling was carried out in February 2012 and November 2012 (4 and 3 months after fertilizer application, respectively) to assess the reproducibility of the observations.

### DNA extraction and pyrosequencing

Total dsDNA was extracted from soil and rhizosphere samples using Mo Bio PowerSoil DNA isolation kits following manufacturer's instructions (Mo Bio Laboratories, Inc., Carlsbad, CA, USA). To profile fungal communities, the fungal internal transcribed spacer (ITS) region was PCR-amplified from isolated soil and rhizosphere DNA using ITS1F (5'- CTTGGTCATTTAGAGGTAA-3') and ITS2R (5'-GCTGCGTTCTTCATCGATGC-3') primers modified on the 5' end to contain the 454 FLX Titanium Lib L adapters A and B, respectively. The forward and reverse primer contained a 10-base multiplex identifier (MID) barcode sequence between the primer target sequence and the adapter. A unique MID was used for each sample to identify sequencing reads to sample. PCR was performed using AmpliTaq Gold 360 master mix (Applied Biosystems). Thermocycling conditions were as follows: 95°C for 5 min; 30 cycles of 94°C for 30 s, 55°C for 45 s, 72°C for 60 s; 72°C for 7 min. Amplicons were purified using Ampure magnetic beads (Beckman Coulter), quantified using Picogreen (Invitrogen) fluorometry on the Quant Studio (Life Technologies) and normalized to 1 × 10^9^ molecules/μL. Normalized samples were set up for qPCR (KAPA Biosystems kit) on the Quant Studio and then normalized and pooled for 454 sequencing. Sequencing was performed by the Australian Genome Research Facility Ltd.

### Bioinformatics

The Quantitative Insights Into Microbial Ecology (QIIME) workflow was implemented for data analysis. Raw data were first de-multiplexed with a quality threshold of 150-bp minimum read length and minimum average quality score of 25. Further flanking regions were trimmed to extract ITS regions using ITSx 1.0.9^30^. Quality filtered reads were clustered to pick operational taxonomic units (OTUs) using a closed reference-based method and then assigned taxonomy using the RDP classifier at a confidence level of 80% with reference to the UNITE database dated Nov 2012 (http://unite.ut.ee/repository.php). The resulting OTU table was used to estimate fungal diversity within (α-diversity) and between samples (β-diversity). Fungal species richness was represented using the Chao1 metric and Simpson's index. The binary Chord's metric was used to compare microbial communities based on their composition. Analyzed data were visualized by three-dimensional Principal Component Analysis (PCA).

### Nucleotide sequence accession number

Pyrosequencing data were deposited in European Nucleotide Archive (ENA accession ERA372942).

## Figures and Tables

**Table 1:** Fifteen fungal OTUs whose relative proportion in the community was increased or decreased the most between low and high N fertilizer dose. Ratio denotes the relative abundance of OTUs in low N compared to high N. The samples were from February 2012 collection

Rhizosphere					Soil				
OTU	Genus	Function	Ref	Ratio	OTU	Genus	Function	Ref	Ratio
110	*Clonostachys*	Biocontrol	31	41.8	175	Unidentified	n/a		21
178	*Agrocybe*	Decomposer	32	26.3	110	*Clonostachys*	Biocontrol	31	14.2
123	*Emericellopsis*	Biocontrol	33	26	82	*Calcarisporiella*	n/a		14
205	unidentified	n/a		24.3	207	*Waitea*	n/a		13.3
183	*Clitopilus*	Biocontrol	34	23.7	199	*unidentified*	n/a		13
212	*Resinicium*	Decomposer, biocontrol	26,35	16.2	43	*Epicoccum*	Biocontrol	36	11.3
145	*Kananascus*	n/a		11.8	253	unidentified	n/a		10.2
180	*Conocybe*	Biocontrol	37	11.2	250	unidentified	n/a		7.7
126	*Sarocladium*	Pathogen	38	7.3	151	*Scedosporium*	n/a		6.7
154	*Corynascus*	Decomposer	39	6	243	unidentified	n/a		6.6
199	unidentified	n/a		5.5	180	*Conocybe*	Biocontrol	37	6.6
196	unidentified	n/a		5.3	205	unidentified	n/a		5.7
204	*Ceratobasidium*	Biocontrol	40	5.3	216	unidentified	n/a		5.3
1	*Aplosporella*	n/a		4.9	97	*Dactylella*	Biocontrol	41	5.3
67	*Emericella*	Pathogen	42	4.6	76	*Spiromastix*	n/a		3.8
136	*Nectria*	Pathogen	43	0.03	196	unidentified	n/a		0.04
160	*Zopfiella*	n/a		0.03	89	*Scolecobasidium*	n/a		0.04
48	*Preussia*	Biocontrol	44	0.04	129	*Trichothecium*	Pathogen	45	0.05
161	unidentified	n/a		0.07	126	*Sarocladium*	Pathogen	38	0.07
113	*Cordyceps*	Biocontrol	46	0.07	258	*Mortierella*	Biocontrol	47	0.09
4	*Capnodium*	Pathogen	48	0.15	200	unidentified	n/a		0.10
194	*Coprinopsis*	n/a		0.17	197	*Psilocybe*	n/a		0.10
114	*Metarhizium*	Biocontrol	49	0.17	143	*Arthrinium*	Biocontrol	50	0.12
108	unidentified	n/a		0.18	187	unidentified	n/a		0.14
93	*Scytalidium*	n/a		0.21	186	*Vascellum*	n/a		0.20
44	*Exserohilum*	Decomposer, biocontrol, pathogen	51	0.21	64	*Thermoascus*	Decomposer	52	0.20
97	*Dactylella*	Biocontrol	41	0.22	122	*Acremonium*	n/a		0.20
236	*Dioszegia*	n/a		0.23	236	*Dioszegia*	n/a		0.22
59	*Rhinocladiella*	n/a		0.25	111	unidentified	n/a		0.22
140	*Ophiocordyceps*	Biocontrol	53	0.27	191	*Pluteus*	n/a		0.24

**Table 2:** Fifteen fungal OTUs whose relative proportion in the community was increased or decreased the most between low and high N fertilizer dose. Ratio denotes the relative abundance of OTUs in low N compared to high N. The samples were from November 2012 collection

Rhizosphere					Soil				
OTU	Genus	Function	Ref	Ratio	OTU	Genus	Function	Ref	Ratio
183	*Clitopilus*	Biocontrol	34	122.9	178	*Bolbitius*	n/a		388.4
208	*Resinicium*	Decomposer, Biocontrol	26,35	44.9	202	*Ceratobasidium*	Biocontrol	40	79.1
189	*Cyathus*	Decomposer, Biocontrol	54,55	34.5	179	*Conocybe*	Biocontrol	37	35.2
33	unidentified	n/a		29.2	103	*Clonostachys*	Biocontrol	31	24.6
202	*Ceratobasidium*	Biocontrol	40	26.6	197	unidentified	n/a		17.8
103	*Clonostachys*	Biocontrol	31	23.3	89	*Dactylella*	Biocontrol	41	16.2
211	unidentified	n/a		17.5	108	*Cordyceps*	Biocontrol	46	15.0
230	unidentified	n/a		9.8	81	*Retroconis*	n/a		13.5
219	unidentified	n/a		6.0	83	*Xylogone*	Biocontrol	56	11.8
204	unidentified	n/a		4.6	211	unidentified	n/a		11.5
182	*Gymnopilus*	Decomposer	57	4.5	187	*Marasmius*	n/a		9.5
11	*Septoria*	Pathogen	58	4.5	239	*Spizellomyces*	Decomposer	59	8.8
218	unidentified	n/a		4.4	106	*Beauveria*	Biocontrol	60	7.1
40	*Epicoccum*	Biocontrol	36	4.3	61	*Emericella*	Biocontrol	33	5.8
187	*Marasmius*	n/a		3.8	22	*Periconia*	Pathogen	61	5.0
146	*Verticillium*	Pathogen	62	0.03	205	*Rhizoctonia*	Pathogen	63	0.02
186	Unidentified	n/a		0.04	177	*Agrocybe*	Decomposer	32	0.04
125	*Sarocladium*	Pathogen	38	0.05	29	unidentified	n/a		0.08
147	*Microascus*	n/a		0.07	196	*Lepista*	n/a		0.08
25	*Coniothyrium*	Pathogen	64	0.08	105	unidentified	n/a		0.08
194	unidentified	n/a		0.09	192	*Coprinopsis*	n/a		0.11
156	*Cladorrhinum*	Biocontrol	65	0.09	62	*Eupenicillium*	Biocontrol	66	0.18
102	*Bionectria*	Biocontrol	67	0.10	147	*Microascus*	n/a		0.23
50	*Cyphellophora*	n/a		0.10	92	*Meyerozyma*	Biocontrol	68	0.24
68	*Talaromyces*	Biocontrol	69	0.13	12	unidentified	n/a		0.27
34	*Phaeosphaeriopsis*	Pathogen	70	0.14	122	*Emericellopsis*	Biocontrol	33	0.27
90	*Dactylellina*	Biocontrol	71	0.16	194	unidentified	n/a		0.28
245	unidentified	n/a		0.16	185	*Vascellum*	n/a		0.28
57	unidentified	n/a		0.17	121	*Acremonium*	n/a		0.29
45	*Preussia*	Biocontrol	44	0.17	167	*Nigrospora*	Pathogen	72	0.32
